# Synthesis of Porous Activated Carbon Doped with Tetramethylammonium Hydroxide: Evaluation of Excellent Gasoline Vapor Adsorption Performance and Activation Mechanism

**DOI:** 10.3390/molecules28155868

**Published:** 2023-08-04

**Authors:** Chenyu Wu, Jing Yang, Yu Gong, Yongming Ju, Jiahui Tao, Xinmeng Jiang

**Affiliations:** 1 School of Geographical Science, Nantong University, Nantong 226019, China; chenyuw525@outlook.com (C.W.); tao2912677204@outlook.com (J.T.); jxinm109@outlook.com (X.J.); 2 Nanjing Institute of Environmental Sciences, Ministry of Ecology and Environment (MEE), Nanjing 210042, China; gongyu@nies.org; 3 South China Institute of Environmental Sciences, Ministry of Ecology and Environment (MEE), Guangzhou 510655, China

**Keywords:** activated carbon, activation mechanism, adsorption, gasoline vapor, tetramethylammonium hydroxide

## Abstract

The rapid urbanization and industrialization in China have led to an urgent dilemma for controlling urban air pollution, including the intensified emission of gasoline vapor into the atmosphere. Herein, we selected highland barley straw as a raw material and KOH and tetramethylammonium hydroxide (TMAOH) as activators to synthesize nitrogen-doped layered porous carbon (K-thAC) by a three-step activation method. The obtained K-thAC materials had a high specific surface area, reaching 3119 m^2^/g. Dynamic adsorption experiments demonstrated a superior adsorption capacity of up to 501 mg/g (K-thAC-25) for gasoline vapor compared with other documented carbon adsorbents. Moreover, adjusting the ratio of raw materials with a series of active ingredients could further improve the pore properties of the obtained K-thACs and their adsorption performance for gasoline vapor. Furthermore, the K-thAC materials were also characterized by Fourier transform infrared spectroscopy (FTIR), scanning electron microscopy (SEM), synchronous thermogravimetry (STA), X-ray powder diffraction (XRD), energy dispersive spectroscopy (EDS), X-ray photoelectron spectroscopy (XPS), and nitrogen adsorption tests. This study synthesized a novel plant-based material to treat gasoline vapor pollution efficiently.

## 1. Introduction

Volatile organic compound (VOC) emissions into the atmosphere have direct neurotoxic and carcinogenic impacts on people’s health along with greenhouse effects, photochemical smog, and stratospheric ozone depletion [1,2,3]. Coal combustion, vehicle emissions, the coking industry, biomass combustion, and oil volatilization are the main anthropogenic sources of urban VOC emissions [4,5]. Gasoline vapor accounts for approximately 10% of the total VOC emissions in famous cities and regions around the world (Table S1). The main sources of gasoline vapor are the volatilization of gas stations and escape from vehicle fuel tanks [2]. In research on gasoline stations, 24% of air samples at station boundaries showed non-methane hydrocarbon (NMHC) concentrations over the national standard of 4 mg/m^3^ [2]. Notably, a benzene concentration of 1620 ppb has been reported [6], which is 10 times higher than at other common monitoring points. A large amount of gasoline vapor could cause serious damage to the health of gas station staff and urban residents. Health risk assessment results indicate that the residents living in areas with relatively high gasoline vapor concentrations have a higher increment lifetime cancer risk (ILCR) than residents in other low-concentration areas [7]. Accordingly, it is urgent to eliminate the emissions of gasoline in urban areas.

Presently, gasoline, known as a complex VOC, has mainly been treated by adsorption (including adsorption using organic solvents, adsorption onto porous materials, and condensation recovery methods) [8]. One of the most widely used methods is adsorption by porous materials owing to the highly efficient and relatively easy obtainment process, making it suitable for widespread use. Based on the comprehensive consideration of the average specific surface area, pore capacity, and VOC adsorption capacity, different adsorbent materials are ranked as follows: metal organic skeletons (MOFs) > activated carbons (ACs) > super cross-linked polymeric resins (HPRs) > molecular sieves [9]. MOFs usually require complex synthesis processes and high synthesis costs [10]. ACs have apparent manufacturing cost advantages compared to MOFs. To the best of our knowledge, ACs are regarded as one of the most widely used adsorbents due to their excellent properties, including large specific surface area, rich functional groups, good chemical stability, high mechanical strength, acid and alkali resistance, and good recyclability [11]. Nevertheless, it is still a great challenge to synthesize affordable, effective, and sustainable VOC-treatment adsorbents.

Highland barley is the main food crop in Tibet, representing the largest area under cultivation and field production. It is a rare crop that can grow at high altitudes of 4200–4500 m [12]. Highland barley cultivation results in the massive production of highland barley straw, which may be an important biological source for the production of ACs [13,14]. Barley straw has a similar I-shaped structure and cavity to bamboo, which makes carbonization more uniform during carbon production [15]. Moreover, the performance and specific applications of ACs are substantially dependent on the preparation conditions, porosity characteristics, surface chemistry, and modifications. The currently available activation methods for general ACs mainly include acid, alkali, salt, and steam activation. To the best of our knowledge, it is more appropriate to use alkaline materials to adsorb gasoline vapor, which is typically composed of C_5_ to C_12_ aliphatic hydrocarbons and cycloalkanes. Alkaline treatment could improve the VOC adsorption capacity due to the increase in specific surface area, pore volume, and hydrophobicity, as well as the reduction in the total oxygen functional groups. ACs surfaces treated with a strong alkaline are rough and purified, and different pore sizes are observed on the surface and inside the obtained particles. Notably, potassium ions are able to activate carbon to generate more mesopores and macropores within the range of 20–50 nm compared to sodium ions, and this could be beneficial for the diffusion of targeted gas molecules [16,17]. Olivine-residue-based AC modified with KOH revealed a specific surface area of 2387 m^2^/g at high temperature (850 °C) [18]. Similarly, KOH activators can synthesize porous ACs with specific surface areas of 2326.5 m^2^/g and 2290 m^2^/g, respectively [19,20]. Therefore, KOH is regarded as one of the most effective activating agents for the alkali modification of ACs.

Tetramethylammonium hydroxide (TMAOH), functioning as an organic strong-alkaline-based etching agent for ACs, could sufficiently remove the residual oxygenated acidic functional groups owing to its abundant electrons. TMAOH has been used to isolate high-molecular-weight and high-yield hemicellulose from poplar holocellulose at room temperature [21]. About 90% of the hemicellulose could be dissolved at room temperature within 1 h. Compared with NaOH solution fractionation, the hemicellulose separated by the TMAOH solvent possessed a more complete structure and higher purity. In addition, the retention of cellulose after TMAOH treatment rose to 90.2%, and the crystal structure of the cellulose in the residue remained essentially unchanged [21]. Accordingly, TMAOH is a key activator for purifying the surface of ACs and introducing functional groups. It could function as an efficient nitrogen doping agent. Accordingly, we herein innovatively selected TMAOH as a nitrogen-containing functional group introduction agent and attempted to explore its activation mechanism and advantages. This study provides a feasible method for preparing high-performance ACs with new precursors.

## 2. Results and Discussion

### 2.1. Effects of Activation Temperature, Time, and Activator Dosage

Based on the static adsorption rate, the optimal K-AC was selected by changing the activation temperature and reagent ratio. Compared to the 850 °C AC, the 750 °C AC showed less fully developed pores, some of which were still clogged by coal tar and other substances. As for the 900 °C AC, severe micropore corrosion caused a rise in macropores, a drop in specific surface area, and a reduction in adsorption capacity. Overall, the adsorption rate of the ACs increased with the increase in temperature and carbon/alkali ratio. However, a high temperature and high carbon alkali ratio led to a lower carbon yield. Therefore, in order to consider efficiency, energy consumption, and activator consumption, we constructed the following equation to measure the comprehensive performance of the AC:

(1) $$\omega =1000\frac{k\alpha }{T}\;$$

where ω (s^−1^) represents the comprehensive performance, k (unitless) represents the static adsorption rate of the corresponding ACs at 120 min, α is the carbon/alkali ratio, T (s) is the corresponding activation temperature, and 1000 is a constant.

Table 1 shows the adsorption rate under different conditions and the ω values calculated by Equation (1). The ω values indicated that the most effective combination of activation temperature and carbon/alkali ratio was 850 °C and 1:5. Hence, the following experiments were carried out under the above conditions for K-thACs synthesis.

### 2.2. Dynamic Adsorption Test and Static Adsorption Test

**Figure 1:**
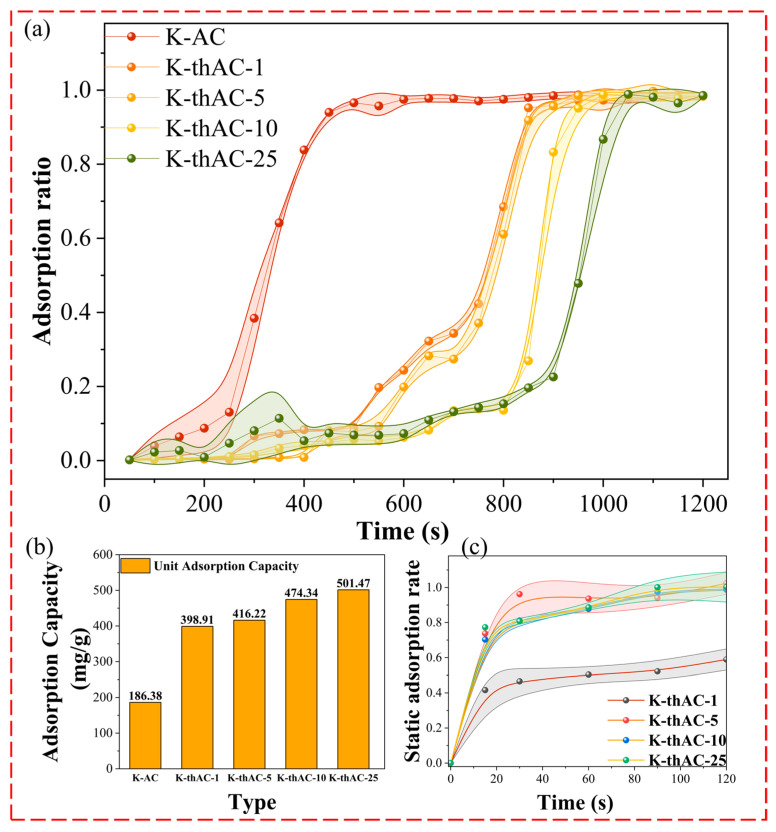
(**a**) Dynamic adsorption breakthrough curve; (**b**) Total adsorption capacity of K-thACs; (**c**) Static adsorption curve.

We took a C/C_0_ ratio of 20% as the breakthrough point for adsorption. The breakthrough times of K-thAC-1, K-thAC-5, K-thAC-10, and K-thAC-25 were 550, 600, 800, and 850 s, as shown in Figure 1a, but the breakthrough time of K-AC was only 250 s. The results showed that K-thAC-25 had good adsorption performance for gasoline vapor, and the activation effect of TMAOH was obvious. Breakthrough measurement as a direct method is widely used to evaluate dynamic adsorption performance [22]. Thus, the adsorption breakthrough curves of the K-thACs reflected the adsorption performance of the ACs for gasoline vapor. The outlet gas concentration over time is shown in Figure 1a. In other studies, there has also been a small upward slope at the start of the adsorption breakthrough curve [23], which can be attributed to the influence of mass transfer resistance [24]. The curves have shown important slope changes indicating changes in the accessibility of the pores and interactions with the carbon surface [18].

Before the C/C_0_ reached 10%, the breakthrough curve was approximately a straight line. We herein speculated that the gasoline vapor was almost adsorbed by the carbon materials during this period. Within the 10% to 20% stage of the C/C_0_, the outlet concentration of gasoline vapor gradually increased after penetration. After the C/C_0_ reached 20%, the concentration of gasoline vapor at the outlet rose rapidly until a stable stage. Generally, the sorption capacity increased as the specific surface area and pore volume increased. The specific surface area had a significant effect on the breakthrough time and adsorption capacity. Additionally, the textural characteristics of the material, such as the pore volume, microporosity, and hierarchical porous structure, impacted the adsorption procedure. Compared to K-AC, the dynamic adsorption rate increased significantly after TMAOH activation. This indicated that the introduction of functional groups using TMAOH obviously improved the adsorption rate.

The graph in Figure 1b was calculated using Equation (13). Based on Figure 1b, we could see a significant increase in the adsorption capacity of the ACs after the use of TMAOH, with the total adsorption capacity being approximately twice that of K-AC; the total predicted sorption was 501 mg/g over 1200 s. Moreover, the total adsorption capacity slowly increased as the concentration of TMAOH increased. According to Figure 1a, the activation by TMAOH obviously prolonged the breakthrough time, and further increasing the TMAOH concentration positively affected the adsorption performance.

The static adsorption curve of gasoline vapor is shown in Figure 1c. All K-thACs basically reached the adsorption saturation state at 20 min. All K-thAC samples exhibited type IV isotherms. Due to the rich microporous structure of the K-thACs, the adsorption capacity for gasoline vapor rapidly increased at low concentrations. As the concentration further increased, the adsorption capacity for gasoline vapor gradually reached an adsorption equilibrium, and multi-layer adsorption could occur in the pores of the sample [25].

### 2.3. Characterization

**Figure 2:**
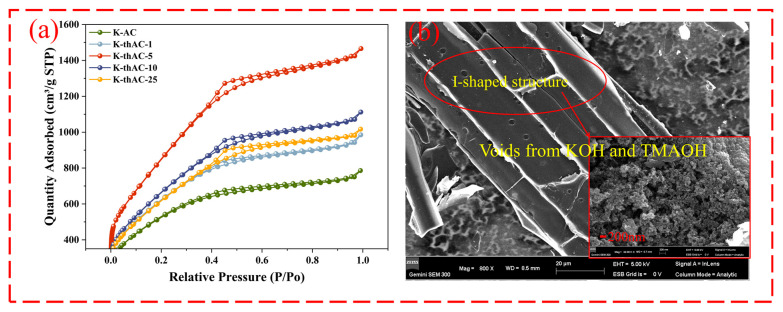
(**a**) Nitrogen adsorption/desorption isotherm; (**b**) I-shaped structure.

The nitrogen adsorption/desorption isotherms and pore distribution of the various adsorbents are shown in Figure 2a. The structural parameters are shown in Table 2. According to the IUPAC classification, the isotherms are similar to the type I isotherms and have microporous carbon characteristics. In addition, H4 hysteresis loops could be observed, indicating the simultaneous existence of microporous and mesoporous structures [26]. The initial shape of the isotherms indicated monolayer coverage and, therefore, the presence of hysteresis loops at P/P_0_ > 0.4 indicated the presence of microporosity. The surface areas of K-AC, K-thAC-1, K-thAC-5, K-thAC-10, and K-thAC-25 were calculated as 1871.21, 2246.07, 3119.43, 2435.61, and 2243.78 m^2^/g, respectively. The comparison of surface area values indicated that the addition of TMAOH further developed the surface area and pore volume of the original carbon. However, the surface area did not increase with the increase in the concentration of TMAOH. Instead, the optimal specific surface area was obtained at a concentration of 5%. This phenomenon could be explained by the main role played by TMAOH at different concentrations [27,28]. At lower concentrations (<5%), the main role of TMAOH was as an etchant, further expanding the pore volume of carbon under mild conditions. At higher concentrations (>10%), the main function of TMAOH became the introduction of functional groups, which occupied the pore volume and began to reduce the surface area of the carbon. In addition, no structural damage or decrease in the total pore volume of the carbon skeleton caused by TMAOH was observed. The combination of a decrease in average pore size and a unique hysteresis loop may indicate the formation of irregularly shaped and widely distributed internal voids. This could be verified from the SEM images in Figure 2b.

Figure S1 in Supplementary Materials shows the pore distribution of K-thACs. The pore size distribution of the adsorbent was unimodal, concentrated at 2 nm, indicating that the adsorbent contained a large number of micropores. The dynamic diameter of the VOC molecules was also 2 nm, indicating that K-thACs may show a good adsorption effect on VOCs [29].

**Figure 3:**
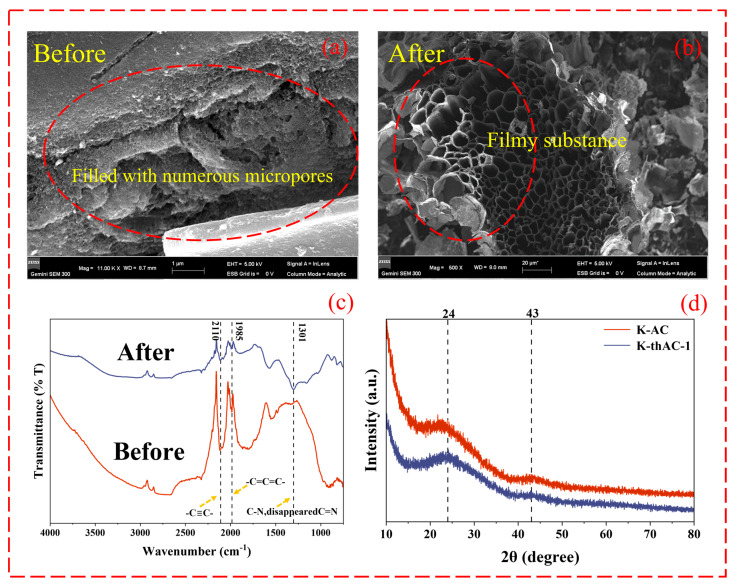
(**a**) The SEM images of K-thAC-10; (**b**) A white film was attached to the carbon pores after adsorption; (**c**) The FTIR images of K-thAC-1 after adsorption; (**d**) The XRD images of K-AC and K-thAC-1.

As shown in Figure 3a,b, the etching of K-AC by KOH and TMAOH resulted in a large number of pores with diameters below 200 nm on the AC, among which even smaller pores with a diameter of around 2 nm could be observed. The number of micropores with diameters below 2 nm is the most important factor affecting the adsorption performance of various adsorbents. When the micropore size of an AC is similar to the dynamic diameter of some VOCs, the micropores have greater adsorption energy [29]. Figure 3a shows that a large number of pore structures appeared in the AC at low magnification. In addition to using KOH, we also considered using TMAOH to further expand the pores. Due to the mild reaction temperature, mesopores and micropores were greatly developed. Research has shown that the mesopores on ACs also have an impact on the adsorption of VOCs. They promote adsorption by accelerating molecular diffusion, so an abundance of micropores helps increase the adsorption capacity [30]. Figure 3b shows the appearance of a thin-film-like substance on the surface of the AC after the adsorption of gasoline vapor, which was ascribed to the adsorbed gasoline vapor.

The functional groups on the surface of K-thAC-1 underwent considerable modifications following adsorption testing, as seen in the FTIR image of Figure 3c. For example, there was an enhanced R-C≡C-R functional group at 2110 cm^−1^ and a -C=C=C- accumulation double bond region at 1985 cm^−1^ [31]. The addition of several non-polar organic compounds by the substantial adsorption of gasoline vapor possibly caused an increase in the homogeneity of the electron clouds on the surface functional groups. The major peak of 1301 cm^−1^ could be assigned to the presence of the linkages of the triazine ring system in N-doped products corresponding to sp^2^ C-N and sp^2^ C=N [32]. After adsorption, this peak disappeared, indicating the role of TMAOH in introducing nitrogen-containing functional groups.

The crystallinity of the K-AC and K-thAC-1 samples was investigated using XRD. The XRD patterns (Figure 3d) showed that K-AC and K-thAC-1 had two insignificant broad peaks of different intensities around 2θ = 24° and 2θ = 43°. These two weak diffraction peaks could be attributed to the (002) and (100) planes in the graphitic carbon structure, respectively [33]. For the K-thACs, the intensity of the peak at 2θ = 24° decreased with the introduction of TMAOH. Notably, for K-thAC-1, the broad diffraction peak at 2θ = 43° almost disappeared, indicating that the graphitization of the carbon material decreased with the introduction of TMAOH. These results suggest that the carbon was more amorphous and more conducive to the adsorption of gasoline vapor after the use of TMAOH compared to the materials without TMAOH [17,34].

**Figure 4:**
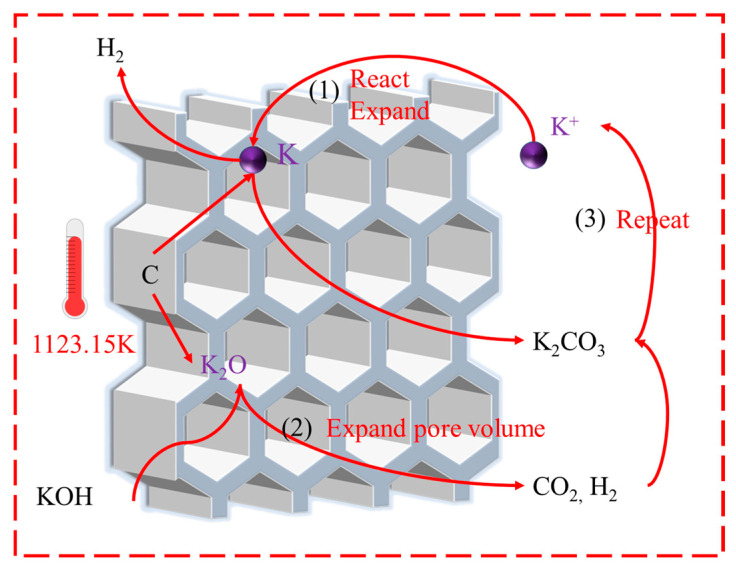
Main reactions during the KOH activation process.

Figure 4 shows the proposed reaction routes between KOH and AC. KOH is a high-quality activator for alkanes and aromatic hydrocarbons that can significantly increase the specific surface area and pore size of ACs [34]. When combined with AC, its main chemical reaction mechanism is ascribed to the reduction of KOH through carbon in an inert gas and a high-temperature atmosphere to produce potassium, as shown in Process (1) in Figure 4, according to Equation (2).

6KOH + 2C→2K + 3H_2_ + 2K_2_CO_3_ (2)

At this point, metal potassium escapes in the form of gas and penetrates into the micropores of the AC [35]. During the reaction process, the entropy of the system increases, which drives the above reaction and other reaction processes corresponding to Process (2) in Figure 4 take place in the meantime, as shown in Equations (3)–(5).

2KOH→K_2_O + H_2_O (3)

C + 2H_2_O→CO_2_ + H_2_ (4)

CO_2_ + K_2_O→K_2_CO_3_ (5)

The gases generated by these reactions further promote the growth of ACs pores. In the third step of Figure 4, some potassium ions re-enter the reaction process, promoting the reactions.

**Figure 5:**
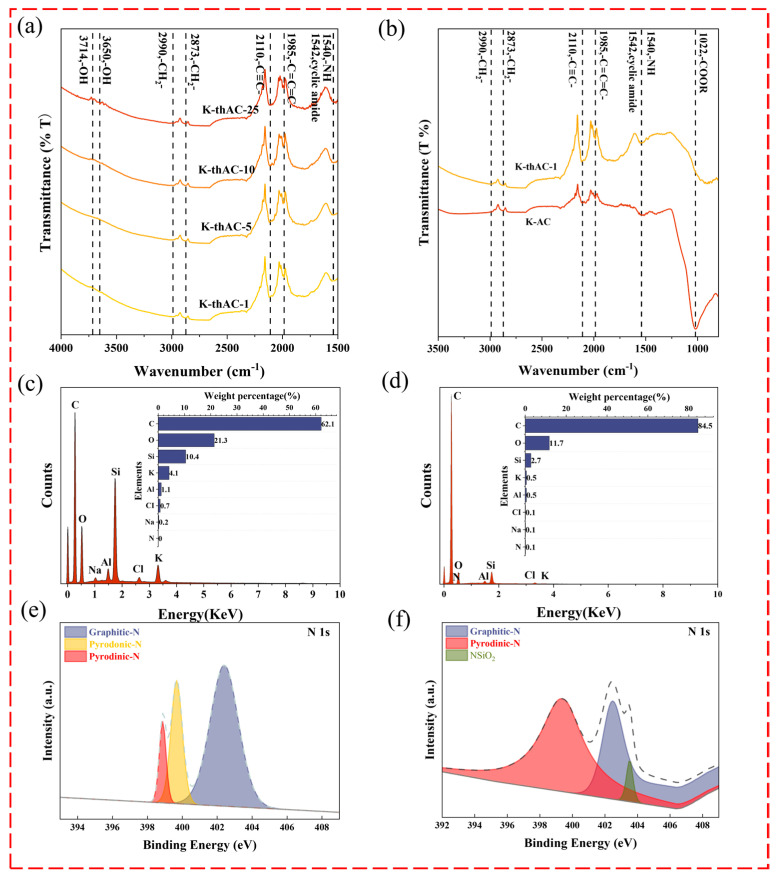
(**a**,**b**) The FTIR images of K-AC, K-thAC-1, K-thAC-5, K-thAC-10, and K-thAC-25; (**c**,**d**) The EDS images of K-AC and K-thAC-1 and the corresponding N elements; (**e**,**f**) The XPS images of nitrogen elements in K-AC and K-thAC-1.

The presence of a large number of tiny peaks in the range of 3600–3800 cm^−1^ can be observed in Figure 5a. The peaks at 3650 cm^−1^ and 3714 cm^−1^ could be attributed to the vibrational stretching of the phenolic hydroxyl group (-OH). Moreover, the aforementioned -OH group, together with the carbon and nitrogen observed at 1542 cm^−1^ [36], could be inferred from the presence of a large number of pyridine groups on the surface of the AC. The carbon also exhibited C-H and C=O absorption peaks at around 1800 cm^−1^, implying the presence of a large number of quinone and ketone groups [31]. Figure 5b exhibits the promotion of the chemical structure modification of ACs by the activation of TMAOH. The enhancement of the R-C≡C-R functional group at 2110 cm^−1^ with the -C=C=C- cumulative double bond region was revealed at 1985 cm^−1^. In the range of 2600 to 3000 cm^−1^, there was an increase in the -CH_2_- functional group from chromene and other carbon-containing functional groups at 2990 cm^−1^ and 2873 cm^−1^ [31,37]. The weakening and disappearance of the ester bond at 1050 cm^−1^ could be attributed to the alkaline hydrolysis of the ester bond in the aqueous TMAOH solution, which is a polar functional group and is not conducive to hydrocarbon uptake. The peak at 1540 cm^−1^ was attributed to -C-NH- [38]. Figure 5c,d show that TMAOH encouraged the introduction of nitrogen-containing functional groups onto the surface of K-thAC-1.

Figure 5e,f show the comparison of the doped N-species in K-thAC-1 before and after adsorption by XPS. The high-resolution N 1s XPS spectra of the N-doped sample could be deconvoluted into three peaks at 399.4 eV, 402.5 eV, and 403.5 eV, which were associated with pyrodinic-N, pyrodonic-N, and graphitic-N, respectively [39,40]. For comparison, K-thAC-1 after adsorption showed significant alteration. In general, pyridinic-N was located at the edge of the graphitic carbon layer, which could efficiently improve the surface alkalinity of the ACs. This also indicated the effective activation via TMAOH. However, the graphitic-N was arranged inside the graphitic carbon layer bonds, contributing less to the interaction with VOCs. The results of the XPS spectra indicated that nitrogen was successfully incorporated into the AC.

In addition, as shown in Figure S2, the desorption temperature of the ACs for gasoline vapor increased from 67 °C to 200 °C compared to carbon without TMAOH activation. It could be inferred that after activation with TMAOH, the chemical interaction between surface functional groups and gasoline vapor molecules made it more difficult to remove the gasoline vapor. The removal of the surface gasoline vapor with the disintegration of the surface functional groups occurred only when the temperature was raised to 200 °C. This also demonstrated the efficiency of the activation technique using TMAOH.

### 2.4. Adsorption Mechanism

#### 2.4.1. Adsorption Isotherm

**Figure 6:**
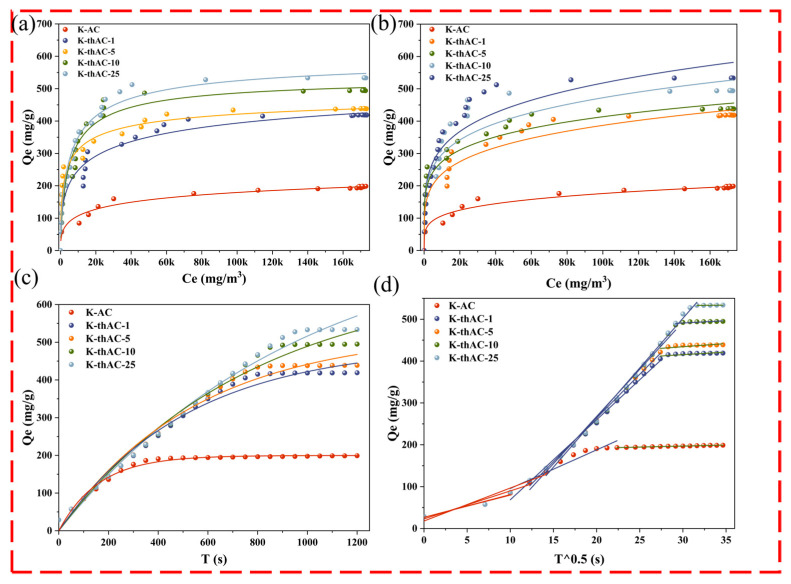
(**a**) Langmuir adsorption isotherm; (**b**) Freundlich adsorption isotherm; (**c**) Pseudo-first-order model; (**d**) Intraparticle diffusion model.

Figure 6a,b show the adsorption isotherms fitted by the Langmuir isotherm and Freundlich isotherm. As shown in Table S2, both models effectively described the sorption process of gasoline vapor onto K-thACs. The different error values for the two methods were calculated using Equations (17)–(19). The reduced chi-square, R^2^, and adjusted R^2^ values for the Langmuir model were all closer to the real situation than those for the Freundlich model. Moreover, the Langmuir isotherm model predicted a maximum equilibrium sorption capacity of 501 mg/g for gasoline vapor from K-thACs. Furthermore, compared with similar studies, the K-thACs revealed a higher sorption capacity than most plant-based ACs, suggesting TMAOH possibly enhanced the sorption capacity.

#### 2.4.2. Adsorption Kinetics Model

According to Fick’s second law, increasing the initial concentration of gasoline vapor could significantly increase the driving force at the interface between the gas molecules and the AC pores. This would promote the effectiveness of collision between the reaction sites on the K-thACs and targeted gas molecules. In order to clearly elucidate the adsorption kinetics, the experimental data were modeled by fitting them to primary and secondary kinetics models and an intraparticle diffusion model, as shown in Figure 6c,d.

Figure 6c indicates that the pseudo-first-order kinetic model could better explain the adsorption than the pseudo-second-order kinetic model (Figure S3). The different R^2^ values for the non-linear methods are shown in Table S4. Of the three different kinetic models, the pseudo-first-order kinetic model and the internal diffusion model had higher R^2^ values.

Figure 6d shows the multicollinearity of the gasoline vapor adsorption on the K-thACs for the second linear cross-section, not from the intercept C across the origin (the value of C is given in Table S4). This suggests that the intraparticle diffusion process is not the only rate-limiting step. Three processes, including film or surface diffusion, intraparticle diffusion, and adsorption–desorption equilibrium, determine the adsorption rate [41]. According to the intraparticle diffusion model, if the adsorption process involves intraparticle diffusion, the curve between q_e_ and t^0.5^ should be linear. If the curve passes through the origin, intraparticle diffusion is the only speed-limiting step [41]. If q_e_ is multilinear for t^0.5^, two or more steps control the adsorption process [42,43].

#### 2.4.3. Adsorption Mechanism

**Figure 7:**
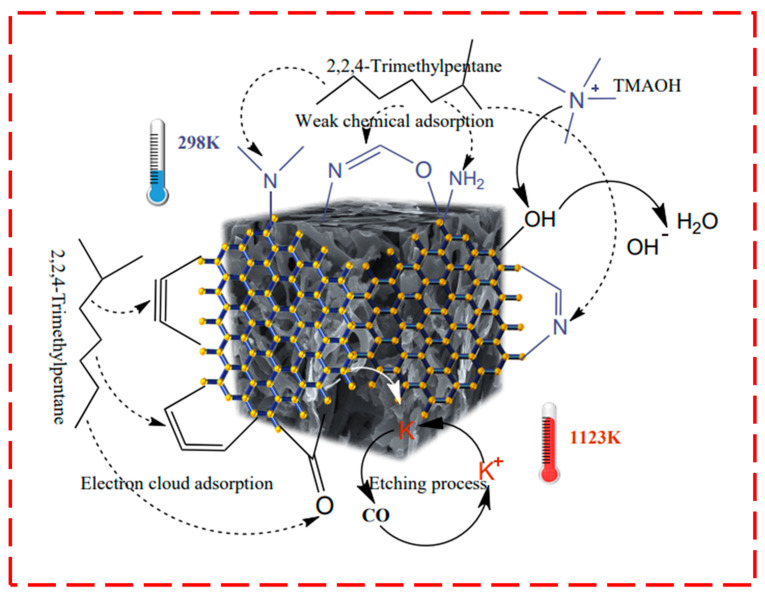
The main chemical changes on the carbon surface.

Figure 7 illustrates how KOH and TMAOH combined to add various basic groups, such as quinone carbonyl and pyranone groups, to the carbon structure. This could be verified by the FTIR plot at 1800 cm^−1^. Due to the presence of a large number of π-bonds and π-π conjugates, these groups could provide a uniform density of electron clouds and reduce the polarity of the obtained AC. The electron-rich region was located in the graphene layer of the AC, which could be found in the EDS patterns shown in Figure 5d, and this region interacted with the π-electrons of the hydrocarbon and aromatic rings [44]. As can be observed from the gasoline adsorption in Figure 1, the change promoted the development of basic and carbonyl groups and significantly improved the adsorption of gasoline vapor.

After activation with TMAOH, the alkalinity on the AC surface apparently increased and further facilitated the insertion of groups like pyrrolidone, chromene, and quinone [31,36]. This resulted in a positively charged AC surface, and π-π bonds could be formed between functional groups, facilitating the adsorption of non-polar gasoline vapor [30]. The p-electron effects and π-π conjugation effects between ACs and gasoline molecules could form weak chemical bonds to initiate chemisorption [45].

The presence of nitrogen-containing functional groups in this study was verified by the EDS images and FTIR at 3500–3800 cm^−1^, respectively. During the adsorption process, TMAOH mainly played a role similar to that of NH_3_ on the carbon surface. The nitrogen-doping mechanism via NH_3_ activation is described by Equations (6)–(8) [16]. Sufficient -OH groups play key roles by reacting with NH_3_ molecules to produce nitrogen functional groups.

R-OH + NH_3_→R-NH_2_ + H_2_O (6)

R-NH_2_ + R-OH→R-N=R + H_2_O (7)

C + NH_3_→C-NH_2_ + C-NH + H_2_ (8)

Similarly to NH_3_ activation, the reactions of the K-thACs are depicted in Figure 7. Ammonia combines with oxygen-containing groups to form amide carbon, which can be converted into different types of nitrogen in the carbon structure [46]. The nitrogen atoms in NH_3_ replace the carbon or oxygen atoms of the intermediate products (C=O, C-O-C, C-OH) in the activation process under the conditions of room temperature and stirring reactions. Therefore, pyridinic nitrogen (-NH_2_), pyrrolic nitrogen (-NH), and graphitic nitrogen (-N=) can be formed, and nitrogen-doping activation can be expressed using Equations (9) and (10).

(CH_3_)_4_N(OH) + R=O+→R=N-R + C(CH_3_)_4_ + H_2_O (9)

(CH_3_)_4_N(OH) + R-O-R→R-N(CH_3_)_3_ + R-O-CH_3_ (10)

In general, TMAOH could efficiently introduce reactive groups onto the AC surface under mild conditions. Moreover, the combination of KOH and the hydroxyl groups of TMAOH functioned to etch pores and introduced electron-rich groups extremely well. Additionally, employing TMAOH for nitrogen doping is more practical and less energy-intensive than other nitrogen doping techniques (such as microorganisms for natural nitrogen-doping protein production [19]).

## 3. Materials and Methods

### 3.1. Materials and Reagents

Highland barley was calcined and activated by a tube furnace (OTF-1200X, Hefei Kejing Material Technology Co., Ltd., Hefei, China). The functional groups on the carbon surface were excited with a digital constant-temperature magnetic stirrer (WS-2A, Changzhou Yuexin Instrument Manufacturing Co., Ltd., Changzhou, China). The adsorption breakthrough curves of the ACs were detected with gas chromatography (ZZ-2800, Jiangsu Zhuozheng Environmental Protection Technology Co., Ltd., Nantong, China). The outlet gas was collected at the same intervals using a gas collection bag and connected to a gas chromatograph for NMHC concentration measurement. We treated NMHC (non-methane hydrocarbon) as the main component of the VOCs in this experiment.

All reagents used in this work were of analytical or guaranteed reagent. Potassium hydroxide (CAS 1310-58-3, KOH) was purchased from Shanghai Titan Technology Co., Ltd., (Shanghai, China); TMAOH (CAS 75-59-2, C_4_H_13_NO, 20% aqueous solution) was purchased from Shanghai Yien Chemical Technology Co., Ltd. (Shanghai, China); and hydrochloric acid (CAS 7647-01-0, HCl) was purchased from China National Pharmaceutical Group Chemical Reagent Co., Ltd. (Shanghai, China) Highland barley was collected from the Qinghai–Tibet region. The barley used (scientific name: *Hordeum vulgare* L. var. *nudum* Hook. f.) was a genus of barley in the family Gramineae, an annual herb with smooth stalks, about 100 cm high, 4–6 mm in diameter, with 4–5 hollow nodes. It is a major food crop in Qinghai–Tibet and is easily available.

### 3.2. Synthesis of Materials

**Figure 8:**
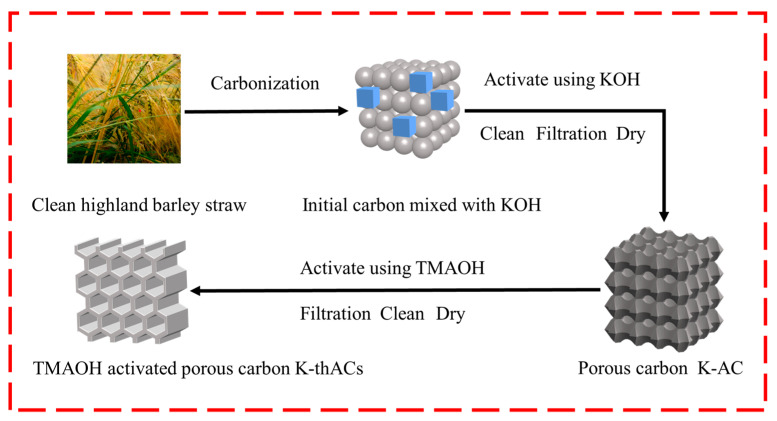
Illustration of the proposed synthesis procedure for K-thACs.

The preparation process of the K-thACs is shown in Figure 8. Based on the common two-step activation method [18], we used a three-step activation method to prepare the ACs to achieve a better adsorption effect.

The barley straw was washed three times with purified water until clean, then naturally dried and cut into 10–15 cm pieces. The barley straw was first carbonized in an argon atmosphere at 500 °C for 2 h. The powdered KOH and initial carbon from the previous step were homogeneously mixed in a 1:5 ratio in an argon atmosphere and activated at high temperatures (350 °C for 2 h, followed by 850 °C for 3 h). The AC was naturally cooled down in an argon environment neutralized with 0.5 N HCl and pure water and then oven-dried (110 °C for 12 h). The sample was designated as K-AC and stored in a sealed bag for use.

### 3.3. Chemical Surface Modification

The K-AC was mixed with TMAOH solution (1%, 5%, 10%, 25%) and stirred at a speed of 1200 r/min for 24 h to introduce surface functional groups. The mixture was filtered to separate the solid, and the K-thACs were neutralized and dried. These samples were labeled as K-thAC-1, K-thAC-5, K-thAC-10, and K-thAC-25.

### 3.4. Characterization of AC

A scanning electron microscope (Gemini SEM 300, ZEISS, Berlin, Germany) was used to take microscopic images of the surface of the ACs. A Fourier transform infrared spectrometer (Nicolet, IS10, Thermo Scientific, Waltham, MA, USA) was used to scan the spectrum at wavelengths from 500 to 4000 cm^−1^. Thermal desorption curves were plotted using a simultaneous thermogravimetric analyzer (STA 449 F3 Jupiter, NETZSCH, Berlin, Germany), and the total oxygen content and the number of different oxygen-containing groups were quantified by the analysis of the TPD profiles. The crystalline phase of the prepared adsorbent was studied by X-ray diffraction (D8 ADVANCE, BRUKER, Billerica, MA, USA). X-ray photoelectron spectroscopy (Nexsa, Thermo Scientific, Waltham, MA, USA) was used to qualitatively detect the presence of nitrogen-containing functional groups. The surface area determination and porosity analysis of the different types of carbon were carried out using an adsorption analyzer (3Flex, Micromeritics, Norcross, GA, USA).

### 3.5. Adsorption Studies

#### 3.5.1. Static Adsorption Test

**Figure 9:**
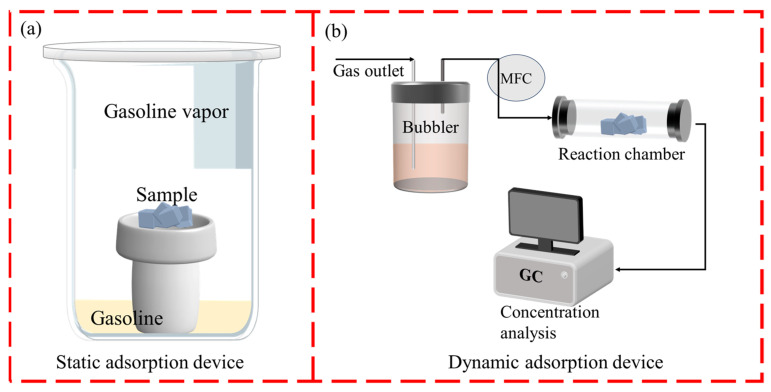
(**a**) Static adsorption experimental device; (**b**) Dynamic adsorption experimental device.

The static adsorption of gasoline vapor was carried out using a glass bottle filled with gasoline vapor at ambient temperature (22–27 °C), with gasoline placed at the bottom of the device (Figure 9a). For each experiment, K-AC (0.1 g) prepared under different conditions was placed on the sample table in the middle of the device. The samples were taken out and weighed every 15 min.

Equation (11) was used to calculate the static adsorption rate of the AC:

(11) $$K_{1}=\frac{m_{1}{\;-\;m}_{0}}{m_{0}}$$

where K_1_ represents the static adsorption rate, m_1_ (g) represents the carbon weight at the corresponding time, and m_0_ (g) represents the carbon weight at the start of the experiment.

#### 3.5.2. Dynamic Adsorption Test

The adsorption ability of K-AC and TMAOH-treated ACs (K-thAC-1, K-thAC-5, K-thAC-10, and K-thAC-25) for static gasoline vapor were studied (Figure 2b). The adsorption of gasoline vapor was carried out at ambient temperature (22–27 °C) using a dry argon gas flow containing gasoline vapor, which was bubbled through a bubbler containing gasoline. For each experiment, the original AC (K-AC) and modified AC (K-thACs) (0.2 g) were placed inside glass tubes with an inner diameter of 1.5 cm. The gasoline vapor flowed through the adsorption tube. All flows were controlled by a mass flow controller (MFC) at a total flow rate of 40 mL min^−1^. The adsorption efficiency was calculated by Equation (12):

(12) $$k_{2}=\frac{C}{C_{0}}$$

where k_2_ represents the dynamic adsorption ratio, C (mg/m^3^) represents the instantaneous adsorption rate at the corresponding time, and C_0_ (mg/m^3^) represents the maximum adsorption amount.

The amount of gasoline vapor absorbed at equilibrium, Q_t_ (mg/g), was determined by Equation (13):

(13) $$q_{e}=\frac{\;40\sum_{n}^{25}{(t}_{n}{\;-\;t}_{n-1}{)\times C}_{n}}{1,000,000}$$

where t_n_ represents the corresponding time (min) at a certain point, C_n_ represents the detection concentration (mg/m^3^) at the corresponding time, 40 is the carrier gas flow rate (mL/min), and 10^6^ is the unit conversion constant.

#### 3.5.3. Kinetics and Equilibrium Studies

In order to understand the kinetics of the adsorption of gasoline vapor by K-thACs, the experimental data were fitted to the nonlinear pseudo-first-order, pseudo-second-order, and intraparticle diffusion models. The pseudo-first-order model was determined by Equation (14):

(14) $${q=q}_{e}{(1\;-\;e}^{{-k}_{3}t})$$

where q_e_ is the quantity of gasoline vapor adsorbed (mg/g) at equilibrium, and k_3_ is the adsorption rate constant (1/min). The values of k_3_ were obtained from the slopes of the linear plots of ln (q_e_ − q_t_) vs. t.

The pseudo-second-order equilibrium adsorption model was given by Equation (15):

(15) $$q=\frac{k_{4}{\mathrm{tq}}_{e}^{2}}{{1+k}_{4}q_{e}t}$$

where k_4_ (g/mg min) is the second-order adsorption rate constant, and q_e_ and k_4_ could then be determined from the slope and the intercept of plot t/qt vs. t.

The intraparticle diffusion model was given by Equation (16):

(16) $${q=q}_{e}{(1\;-\;e}^{{-k}_{5}t})$$

where q_t_ is the quantity of gasoline vapor adsorbed (mg/g) at time t (min); k_5_ represents the adsorption rate (mg/g·min^0.5^); t is the adsorption time; and C is the intercept, representing whether the adsorption process is jointly controlled by the other adsorption stages.

Meanwhile, the theoretical models of Langmuir and Freundlich were also applied to fit the experimental isotherm data. Eventually, the best-fitting model was selected to explain the adsorption kinetics, considering the coefficient of determination (R^2^). In addition to the error function of R^2^, the reduced chi-square values (reduced chi-square) and the adjusted coefficient of determination R^2^ (adjusted R^2^), which were also investigated to determine the best-fitting model, are shown in Equations (17)–(19) [47].

(17) $$\chi_{\mathrm{red}}^{2}=\frac{\chi }{K}$$

(18) $$R^{2}=\frac{\sum_{i=1}^{n}{{(q}_{e,\mathrm{cal}}\;-\bar{{\;q}_{e,\exp}})}^{2}}{\sum_{i=1}^{n}{{(q}_{e,\mathrm{cal}}-\;\bar{q_{e,\exp}})}^{2}{+(q}_{e,\mathrm{cal}}{\;-\;q}_{e,\exp}{)}^{2}}$$

(19) $${\mathrm{Adjusted}\;R}^{2}=\left\{1\;-\;\left[\frac{{(1\;-\;R}^{2})(n\;-\;1)}{\left(n\;-\;k\;-\;1\right)}\right]\right\}$$

where χ is cardinality, k is freedom, q_e,exp_ (mg/g) is the experimental equilibrium adsorption capacity, q_e,cal_ (mg/g) is the calculated adsorption capacity obtained from the models, q_e,exp_ (mg/g) is the average value of q_e,exp_, n is the number of observations in the experimental data, and k is the number of independent variables.

## 4. Conclusions

In this study, we successfully synthesized a series of nitrogen-doped porous carbons (K-thACs) using different ratios of raw materials and active agents (KOH and TMAOH). The main conclusions are summarized as follows:

(1) The K-thACs had a maximum adsorption capacity of 501 mg/g for gasoline vapor, mainly owing to the specific surface area of 3119 m^2^/g. Moreover, the K-thACs showed great advantages compared with most industrial ACs materials for gasoline vapor adsorption.
(2) The mechanism of the adsorption of gasoline vapor by K-thACs could be attributed to the substitution of intermediate carbon or oxygen atoms by nitrogen atoms in TMAOH, which is similar to NH_3_ doping.
(3) The porous structure of the K-thACs could be effectively etched owing to the basicity of TMAOH under mild conditions. This study provided proof of the superiority of TMAOH as a nitrogen-doping agent.

## Figures and Tables

**Table 1:** Static adsorption rate of ACs under different temperature and activator ratio conditions.

T (s)	α	k	Carbon Yield	ω (s^−1^)
750	1:2	0.2370	0.4956	0.16
	1:5	0.5997	0.4746	0.16
	1:7	0.7069	0.1993	0.13
850	1:2	0.2899	0.4229	0.17
	1:5	0.9366	0.5043	0.22
	1:7	1.1968	0.0698	0.20
900	1:2	0.2192	0.6268	0.12
	1:5	0.7288	0.2225	0.16
	1:7	0.9741	0.0521	0.15

**Table 2:** Specific surface area and porosity data of K-AC, K-thAC-1, K-thAC-5, K-thAC-10, and K-thAC-25.

Samples	SBET (m^2^/g)	Pore Volume (cm^3^/g)	Average Pore Size (nm)	Median Pore Width (nm)
K-AC	1871.21	1.219472	2.6068	0.4271
K-thAC-1	2246.07	1.527990	2.7212	0.4367
K-thAC-5	3119.43	2.273983	2.9159	0.4400
K-thAC-10	2435.61	1.725368	2.8336	0.4389
K-thAC-25	2243.78	1.577563	2.8123	0.4354

## Data Availability

The data presented in this study are available upon request from the corresponding author.

## Supplementary Materials

The following supporting information can be downloaded at: https://www.mdpi.com/article/10.3390/molecules28155868/s1, Figure S1: Pore distribution maps of K-AC, K-thAC-1, K-thAC-5, K-thAC-10 and K-thAC-25; Figure S2: Comparison of TG curves before and after adsorption between K-AC and K-thAC-1; Figure S3: The pseudo-second-order adsorption kinetic curve. Table S1: Content of gasoline related vapors in VOCs source analysis in different countries; Table S2: Parameters of adsorption kinetics of gasoline vapor onto K-thACs; Table S3: The pseudo-first-order and pseudo-second-order kinetic parameters of gasoline vapor adsorption on K-thACs; Table S4: Modeled adsorption parameters for intraparticle diffusion of gasoline vapor on K-thACs. References [48,49,50,51,52,53,54] are cited in the supplementary materials.
