# Supplementary material for: Synthesis of Porous Activated Carbon Doped with Tetramethylammonium Hydroxide: Evaluation of Excellent Gasoline Vapor Adsorption Performance and Activation Mechanism
Source: Molecules. 2023 Aug 4;28(15):5868. doi: 10.3390/molecules28155868 (PMC10421261; doi:10.3390/molecules28155868)
Supplement: Supplementary file 1 [file molecules-28-05868-s001.zip › molecules-2496738-supplementary.pdf]

Table S1. Content of gasoline related vapors in VOCs source analysis in different countries.

Country	Source ratio of gasoline to VOCs	Reference
China (Beijing)	11.3%	[1]
China (Harbin)	12.4%	[48]
China (Taiyuan)	30.9‰ (Based on the stable carbon isotope of benzene (δ 13C) Analysis value）	[49]
China (Pearl River Delta)	21.55%	[50]
South Korea (Seoul)	5%-15%	[51]
Japan	4.6%	[52]
United States (California)	31%	[53]
Japan (Tokyo Metropolitan)	9%–16%	[54]
China (South China)	41.4% (Near gas stations)	[9]

Table S2. Parameters of adsorption kinetics of gasoline vapor onto K-thACs.

**Langmuir model**					
Sample	K-thAC-25	K-thAC-10	K-thAC-5	K-thAC-1	K-AC
Reduced Chi-Sqr	2936.73	570.74	972.45	611.18	62.61
R^2	0.90	0.98	0.95	0.96	0.97
Adjusted R^2	0.89	0.98	0.94	0.96	0.97
**Freundlich model**					
Sample	K-thAC-25	K-thAC-10	K-thAC-5	K-thAC-1	K-AC
Reduced Chi-Sqr	6020.25	3567.39	1554.43	918.72	87.56
R^2	0.80	0.87	0.93	0.95	0.97
Adjusted R^2	0.79	0.87	0.92	0.95	0.97

Table S3. The pseudo-first-order and pseudo-second-order kinetic parameters of gasoline vapor adsorption on K-thACs.

Pseudo-first-order dynamic model					
Sample	K-thAC-25	K-thAC-10	K-thAC-5	K-thAC-1	K-AC
Reduced Chi-Sqr	278.20	371.23	284.87	238.53	57.49
R^2	0.99	0.99	0.98	0.98	0.97
Adjusted R^2	0.99	0.98	0.98	0.98	0.97
**Pseudo-second-order dynamic model**					
Sample	K-thAC-25	K-thAC-10	K-thAC-5	K-thAC-1	K-AC
Reduced Chi-Sqr	23845.14	20990.57	14990.45	13321.78	1210.55
R^2	0.14	0.16	0.18	0.19	0.38
Adjusted R^2	0.11	0.12	0.15	0.15	0.35

Table S4. Modeled adsorption parameters for intraparticle diffusion of gasoline vapor on K-thACs.

type	K_d(mg/g. min$^{0.5}$)	C(mg/g)	R^2	K_d(mg/g.min$^{0.5}$)	C(mg/g)	R^2	K_d(mg/g.min$^{0.5}$)	C(mg/g)	R^2
K-AC	5.24	27.05	0.90	7.27	26.54	0.87	1.32	144.12	0.75
K-thAC-1	5.50	26.89	0.89	18.48	-111.98	0.99	2.45	321.69	0.51
K-thAC-5	6.77	23.07	0.89	20.18	-140.54	0.99	2.51	337.58	0.46
K-thAC-10	6.76	23.08	0.89	22.16	-174.88	0.99	3.11	375.56	0.43
K-thAC-25	7.79	18.35	0.89	22.40	-189.04	0.99	9.47	193.03	0.41

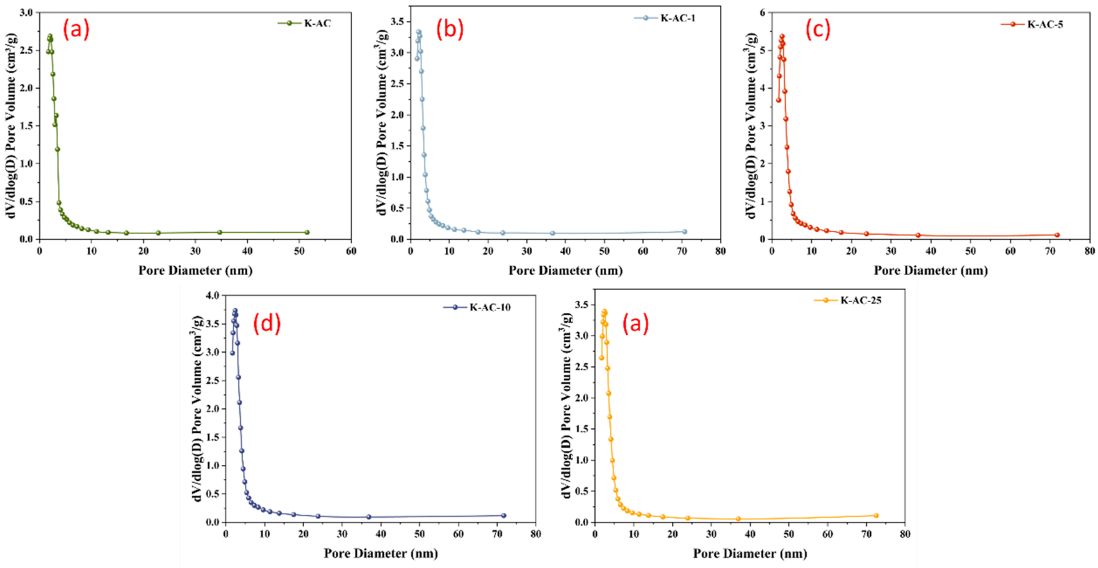

Figure S1. Pore distribution maps of K-AC, K-thAC-1, K-thAC-5, K-thAC-10 and K-thAC-25.

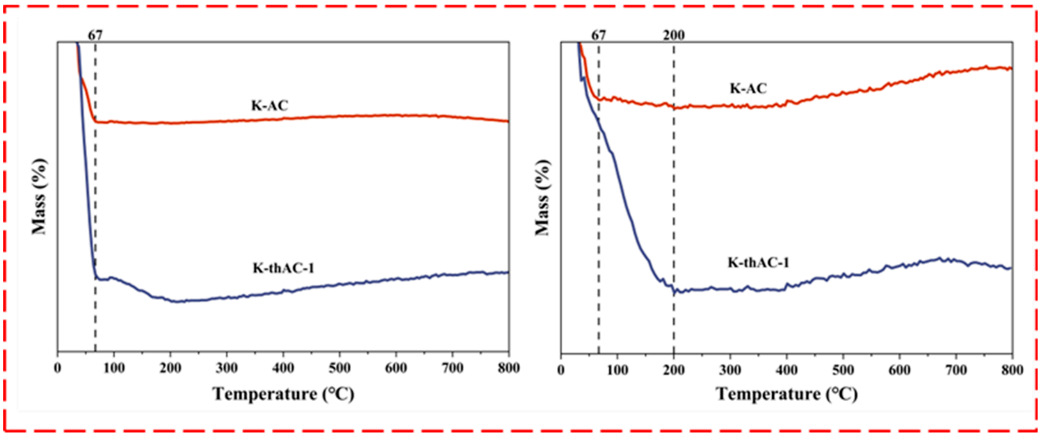

Figure S2. Comparison of TG curves before (left) and after adsorption (right) between K-AC and K-thAC-1.

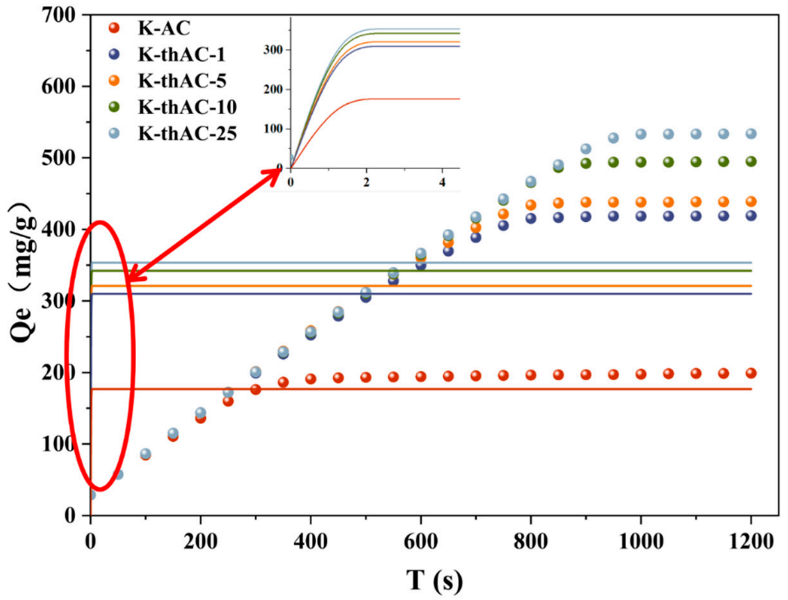

Figure S3. The pseudo-second-order adsorption kinetic curve.
